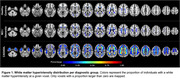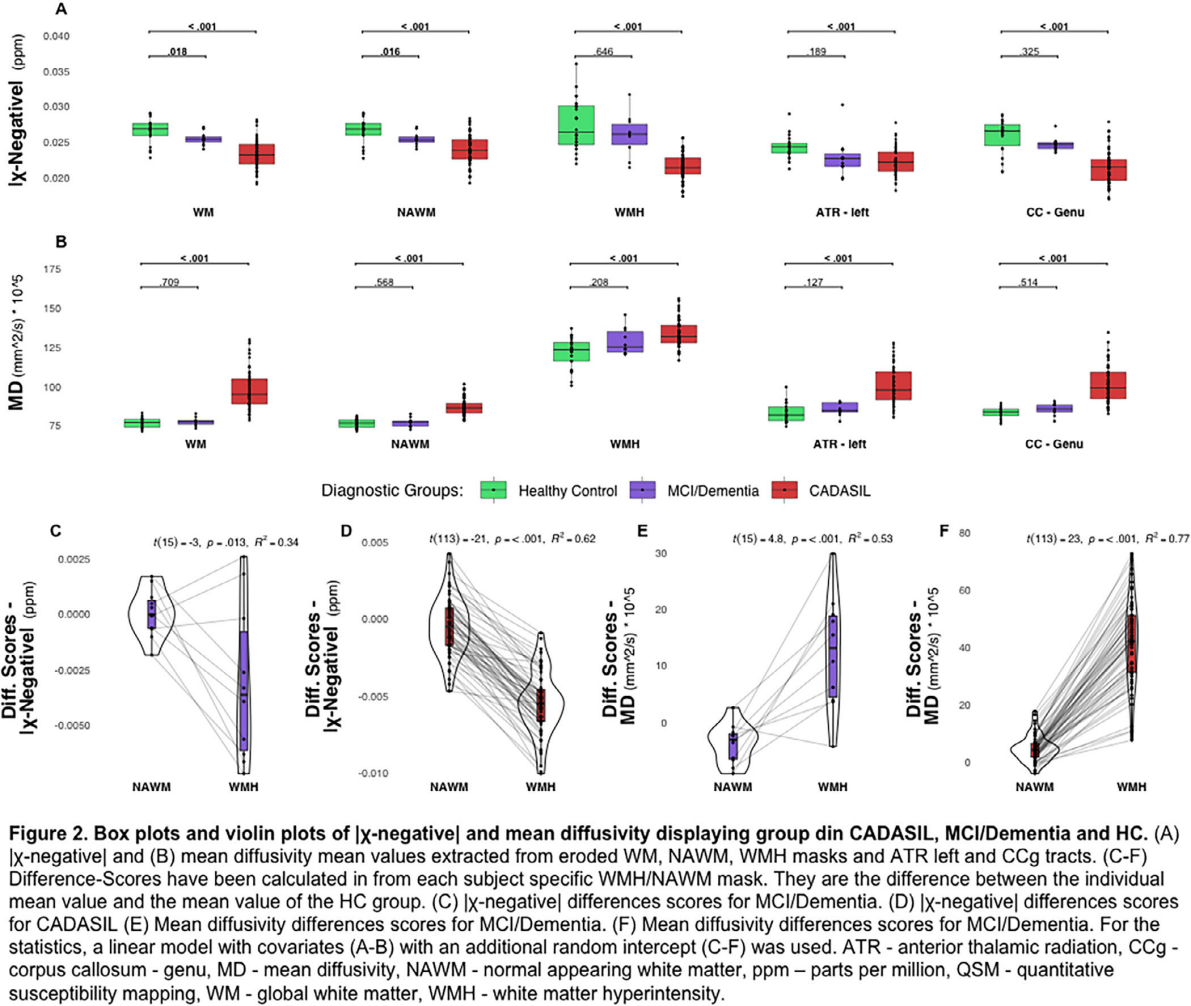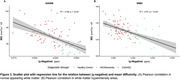# White‐matter myelin is reduced in monogenic small vessel disease and MCI: evidence from MRI source separation

**DOI:** 10.1002/alz.091338

**Published:** 2025-01-09

**Authors:** Jannis Denecke, Anna Dewenter, Jongho Lee, Lukas Pirpamer, Nicolai Franzmeier, Benno Gesierich, Marco Duering, Michael Ewers

**Affiliations:** ^1^ Institute for Stroke and Dementia Research (ISD), University Hospital, LMU, Munich Germany; ^2^ Institute for Stroke and Dementia Research (ISD), University Hospital, LMU, Munich, Bavaria Germany; ^3^ Seoul National University, Seoul Korea, Republic of (South); ^4^ University of Basel, Basel Switzerland; ^5^ Munich Cluster for Systems Neurology (SyNergy), Munich, Bavaria Germany; ^6^ University of Gothenburg, The Sahlgrenska Academy, Institute of Neuroscience and Physiology, Psychiatry and Neurochemistry, Gothenburg Sweden; ^7^ Institute for Stroke and Dementia Research, Ludwig‐Maximilians‐Universität München, LMU München, Munich Germany; ^8^ Medical Image Analysis Center (MIAC) and Department of Biomedical Engineering, University of Basel, Basel Switzerland; ^9^ Institute for Stroke and Dementia Research (ISD), University Hospital, LMU Munich, Munich Germany; ^10^ German Center for Neurodegenerative Diseases (DZNE), Munich, Bavaria Germany

## Abstract

**Background:**

The myelin sheath around axons is of fundamental importance for signal transduction. Myelin is reduced in white matter hyperintensities (WMH), which occur in both small vessel disease (SVD) and Alzheimer’s disease (AD), giving rise to the question to what extent myelin is reduced in these diseases. Here, we employed an advanced MRI based method to assess myelin independently from a major confounding factor, i.e. iron‐related signal, in monogenic small vessel disease (i.e. CADASIL) and in mild cognitive impairment (MCI) & AD dementia.

**Methods:**

We included 62 CADASIL subjects, 11 with MCI or AD dementia, and 22 elderly controls (HC). Using 3D‐T2‐star‐weighted multi‐echo gradient‐echo MRI, we performed susceptibility source separation of diamagnetic (|χ‐negative|, e.g. myelin) and paramagnetic (χ‐positive, e.g. iron) sources (Shin et al., 2021). Mean diffusivity (MD) was calculated from diffusion tensor imaging. We extracted |χ‐negative| and MD values within WMH, normal appearing white matter (NAWM), and two ROIs including the left anterior thalamic radiation and forceps minor (genu) of the corpus callosum, i.e. two strategic tracts for processing speed. Subject‐level difference‐scores between CADASIL or MCI/dementia patients and group‐averaged HC were derived as abnormality scores. Voxel‐based WMH frequencies were mapped for each group. Group differences in |χ‐negative| and MD values were tested using linear regressions, controlled for χ‐positive scores, age, sex, and education.

**Results:**

Voxel‐wise proportions of WMH are mapped for each group in Figure 1. For CADASIL, |χ‐negative| values were decreased, and MD values increased in each ROI compared to the HC group, with intermediate values for the MCI/dementia group (Figure 2 A&B). Worse |χ‐negative| and MD alterations were observed in WMH compared to NAWM (Figure 2 C‐F) in each disease group. Lower |χ‐negative| values were associated with higher MD in WMH (r=‐0.57, Figure 3) and NAWM (r=‐0.56).

**Conclusion:**

|χ‐negative| values showed a marked decrease in WMH of CADASIL patients, suggesting myelin loss in SVD, with less pronounced myelin reduction present in MCI/AD. |χ‐negative| values were only moderately associated with MD, suggesting that each provides complimentary information. Our results encourage future studies to test the cognitive consequences of myelin loss in SVD and MCI.